# Association between Ultrasound Features and the 21-Gene Recurrence Score Assays in Patients with Oestrogen Receptor-Positive, HER2-Negative, Invasive Breast Cancer

**DOI:** 10.1371/journal.pone.0158461

**Published:** 2016-06-30

**Authors:** Eun Young Chae, Woo Kyung Moon, Hak Hee Kim, Won Hwa Kim, Joo Hee Cha, Hee Jung Shin, Woo Jung Choi, Wonshik Han, Dong-Young Noh, Sae Byul Lee, Sei Hyun Ahn

**Affiliations:** 1 Department of Radiology and Research Institute of Radiology, Asan Medical Center, University of Ulsan, College of Medicine, Seoul, Republic of Korea; 2 Department of Radiology, Seoul National University Hospital, Seoul National University College of Medicine, Seoul, Republic of Korea; 3 Department of Surgery, Seoul National University Hospital, Seoul National University College of Medicine, Seoul, Republic of Korea; 4 Department of Surgery, Asan Medical Center, University of Ulsan, College of Medicine, Seoul, Republic of Korea; Fondazione IRCCS Istituto Nazionale dei Tumori, ITALY

## Abstract

A multigene expression assay corresponds to the likelihood of breast cancer recurrence after the initial diagnosis and can be used to guide the decision for additional chemotherapy. However, only few studies have investigated the associations between the imaging features of breast cancer and the results of multigene expression assays. Our study was to identify the relationship between imaging features on ultrasound (US) and the recurrence score (RS) on a 21-gene expression assay in patients with oestrogen receptor (ER)-positive, HER2-negative breast cancer. 267 patients with ER-positive, HER-negative invasive breast cancer who underwent examinations using US and Oncotype DX assay were included. US images were independently reviewed by dedicated breast radiologists who were blind to the RS. Tumour roundness was measured using a laboratory-developed software program. The pathological data were reviewed, including immunohistochemistry results. Univariate analysis was performed to assess the associations between the RS and each variable. Multiple logistic regression analysis was used to identify independent predictors of high RS. Of 267 patients, 147 (55%) had low, 96 (36%) intermediate, and 24 (9%) had high RS. According to the univariate analysis, parallel orientation, presence of calcification in the mass, and tumour roundness were positively associated with high RS. Multiple logistic regression analysis showed that parallel orientation (OR = 5.53) and tumour roundness (OR = 1.70 per 10 increase) were associated with high RS. Parallel orientation and tumour roundness are independent variables that may predict high RS in patients with ER-positive, HER2-negative breast cancer.

## Introduction

It is well established that the biologic characteristics of breast cancer are heterogeneous, complex, and demonstrate distinct intrinsic subtypes that are associated with different responses to treatment and patient outcomes [1,2]. Traditionally, the biological characteristics of breast tumours have been analyzed using histopathology, including immunohistochemistry. Recent developments in genome-wide expression profiling technologies, such as DNA microarrays, provide detailed information for molecular analyses and better classify breast cancers according to their molecular features [3,4]. These advances may provide additional insights for tailored diagnoses, treatments, and surveillance of individual patients.

Oncotype DX (Genomic Health, Redwood City, CA) is a prognostic, profiling, multigene diagnostic assay that estimates the likelihood of disease recurrence in women with early-stage oestrogen receptor (ER)-positive breast cancer [5]. This assay analyzes a panel of 21 genes from a tumour specimen using real-time reverse transcriptase-polymerase chain reaction (RT-PCR) to determine a recurrence score (RS). RS is a number between 0–100 that can be classified into 3 groups: low, intermediate, or high risk. The RS determined by Oncotype DX corresponds to the specific likelihood of breast cancer recurrence within 10 years after the initial diagnosis and can be used to guide the decision for additional chemotherapy. According to the NSABP-B20 trial, adjuvant chemotherapy demonstrates greater benefits toward high-RS tumours, very little benefit toward low-RS tumours, and uncertain benefits toward intermediate-RS tumours [6].

Until now, diagnostic imaging of breast cancer was primarily based on lesion detection, location and disease extent, and monitoring of treatment response. Because diagnostic reference standards have rapidly expanded to the genomic level, there is a need to reestablish the role of imaging in breast cancer. There are several studies on the correlation between the morphological features, histology, and subtypes of breast cancer [7–9]. On breast ultrasound (US), circumscribed margins and posterior acoustic enhancement are associated with high tumour grade or aggressive biology, while spiculated margins and posterior shadowing are correlated with lower tumour grade and better prognosis [10,11]. Quantitative analyses that correlate tumour morphology and the subtypes of breast cancer have also been reported [12]. However, to the best of our knowledge, few studies to date have investigated the associations between the imaging features of breast cancer and the results of multigene expression assays [13,14]. Therefore, the purpose of our current study was to identify the relationship between imaging features on US, RS, and the results of the Oncotype DX gene-expression assay in patients with ER-positive, human epidermal growth factor receptor 2 (HER2)-negative breast cancer. We also assessed if certain US features can be used to distinguish high RS from low or intermediate RS, or low RS from high or intermediate RS, in patients with ER-positive breast cancer.

## Materials and Methods

### Study population

The institutional review board of Asan Medical Center and Seoul National University Hospital approved this retrospective study, and the requirements for informed consent were waived. Patient records or information was anonymized and de-identified prior to analysis. Inclusion was based on the following criteria: 1) diagnosis of ER-positive, HER2-negative invasive breast cancer at 2 institutions; 2) the results of the Oncotype DX gene expression assay were available; and 3) the patient underwent conventional US as part of the preoperative evaluation. Accordingly, we identified 270 women who met the study criteria between August 2010 and April 2014 from two institutions. Of these, 3 patients had undergone excisional biopsy before US, and evaluations of the imaging characteristics were unavailable. Therefore, 267 patients (mean age = 47 years; range = 29–70 years) comprised the study population. 205 patients underwent breast-conserving surgery, and the remaining 62 patients underwent modified radical mastectomy. The interval period between US and surgery was 0–102 days (mean = 17 days). At our institutions, Oncotype DX is recommended for quantifying the likelihood of disease recurrence in patients with node-negative or node-positive ER-positive breast cancers.

### US examinations

Breast US was performed by 12 radiologists (with 1–22 years of experience in breast US) using high-resolution US equipment with a 12–16 MHz linear array transducer (IU22, Philips Medical Systems, Bothell, WA; HDI 5000, Advanced Technology Laboratories, Bothell, WA; LOGIQ 700, GE Medical Systems, Milwaukee, WI; EUB-8500, Hitachi Medical, Tokyo, Japan). Bilateral whole-breast scans were performed as follows: the breast was scanned via the transverse and sagittal orientations, the inner breast was scanned in the supine position, and the outer breast was scanned in the supine oblique position with the patient’s arm raised above her head. We documented the main lesion, including the largest diameter (both horizontal and vertical), and obtained an image perpendicular to its largest respective diameter. We recorded the location in the breast, clockface location to the nearest half-hour, and distance from the nipple.

### Image evaluation

US images were independently reviewed by dedicated breast radiologists with 4 and 3 years of experience interpreting breast US, respectively. Another reader was involved in the interpretation when there was a discrepancy between the readers, and all 3 radiologists reached a consensus. Reviewers were blind to the RS according to Oncotype DX, other imaging results such as mammogram or magnetic resonance imaging, pathologic data, and the other reviewer’s reading.

The US features were described according to the lexicon of the Breast Imaging Reporting and Data System (BI-RADS) [15], which includes the following: mass shape (oval, round, or irregular); orientation (parallel or not parallel); margins (circumscribed, indistinct, angular, microlobulated, or spiculated); echo pattern (anechoic, hyperechoic, complex, hypoechoic, isoechoic, or heterogeneous); and posterior features (none, enhancement, shadowing, or combined). The presence or absence of calcification in the mass and the largest diameter of the lesion in centimeters was also determined.

For the quantitative analysis, tumour roundness was measured using a laboratory-developed software program. The tumour roundness score is a continuous score of how close to a perfect circle. It could be measured on a selected image using the software program. Representative images were digitally transferred from the picture archiving and communications system workstation to a personal computer and processed using the software program [12]. This software allows the researcher to draw lines through, and perimeters around, regions of interest. The software then automatically calculates the perimeter length and area enclosed by a perimeter. Tumour roundness was quantitatively measured by the software developed in-house using Microsoft Visual C++ (version 2005, Microsoft, Redmond, WA) and calculated using the following equation:

$$\text{Tumour roundness}=4\pi \times {\text{A/P}}^{2}$$

where A is the cross-sectional area of the tumour (with the perimeter method)), and P is the measured perimeter length of the tumour.

The tumour roundness score ranges from 1–100 (%). A round-shaped tumour that is a perfect circle would have a roundness score of 100. Tumour roundness was independently assessed by 3 radiologists, and the means of the values were recorded.

### RS and pathological data review

We reviewed RS, which is an output of Oncotype DX. It is a continuous score that is classified into the following categories: low risk (RS < 18), intermediate risk (RS 18–30), and high risk (RS ≥ 31). For the 38 of 267 patients (14%) with multifocal tumors, the tissue for the RS test had been obtained from the largest lesion. The pathological data were also reviewed. The recorded data included lymph node (LN) status, lymphovascular invasion (LVI), histological tumour type, nuclear/histologic tumour grade, and invasive tumour size. Immunohistochemistry (IHC) results, such as progesterone receptor (PR), Ki-67, p53, epidermal growth factor receptor (EGFR or HER1), and cytokeratin (CK) 5/6 status, were also recorded.

### Statistical analysis

Statistical calculations were performed using commercial software (SAS, version 9.3; SAS Institute, Cary, NC). *P*-value < 0.05 was considered statistically significant. Associations between the RS and the categorical variables were assessed using chi-square analysis. Exact tests were used to assess data with low cell frequencies. The Bonferroni correction was applied to address the problem of multiple comparisons and was calculated as the each p value multiplied by the number of comparisons. We summarize the continuous variables using the mean ± standard deviation (SD) and used the 2-sample *t* test to evaluate differences between RS groups. Multiple logistic regression analysis was used to identify the independent predictors of high RS (≥ 31) vs low or intermediate RS (≤ 30), and low RS (< 18) vs high or intermediate RS (≥ 18). Variables with a statistical significance in the univariate analysis were included in the multiple logistic regression analysis and backward elimination was taken to arrive at the final model. We have checked the association among candidate risk or prognostic factors, and there was no violation in the model. Receiver-operating-characteristic (ROC) curve analysis was performed to test the diagnostic performance of distinguishing the high- and low-RS groups.

The correlation between tumour roundness and RS was calculated using Pearson correlation coefficients. The intraclass correlation coefficients (ICC) were calculated to assess inter- and intraobserver variability in tumour roundness, that were determined by the three readers. Fleiss ĸ statistics were used to determine agreement. Note that ĸ = 1.0 denotes perfect agreement, 0.81–0.99 denotes almost perfect agreement, 0.61–0.80 denotes substantial agreement, 0.41–0.60 denotes moderate agreement, 0.21–0.40 denotes fair agreement, and < 0.20 denotes slight agreement [16]. The 2-sided 95% confidence interval (CI) was used to estimate the intraclass correlation coefficient.

## Results

### Association between clinicopathologic data and RS

**Fig 1:**
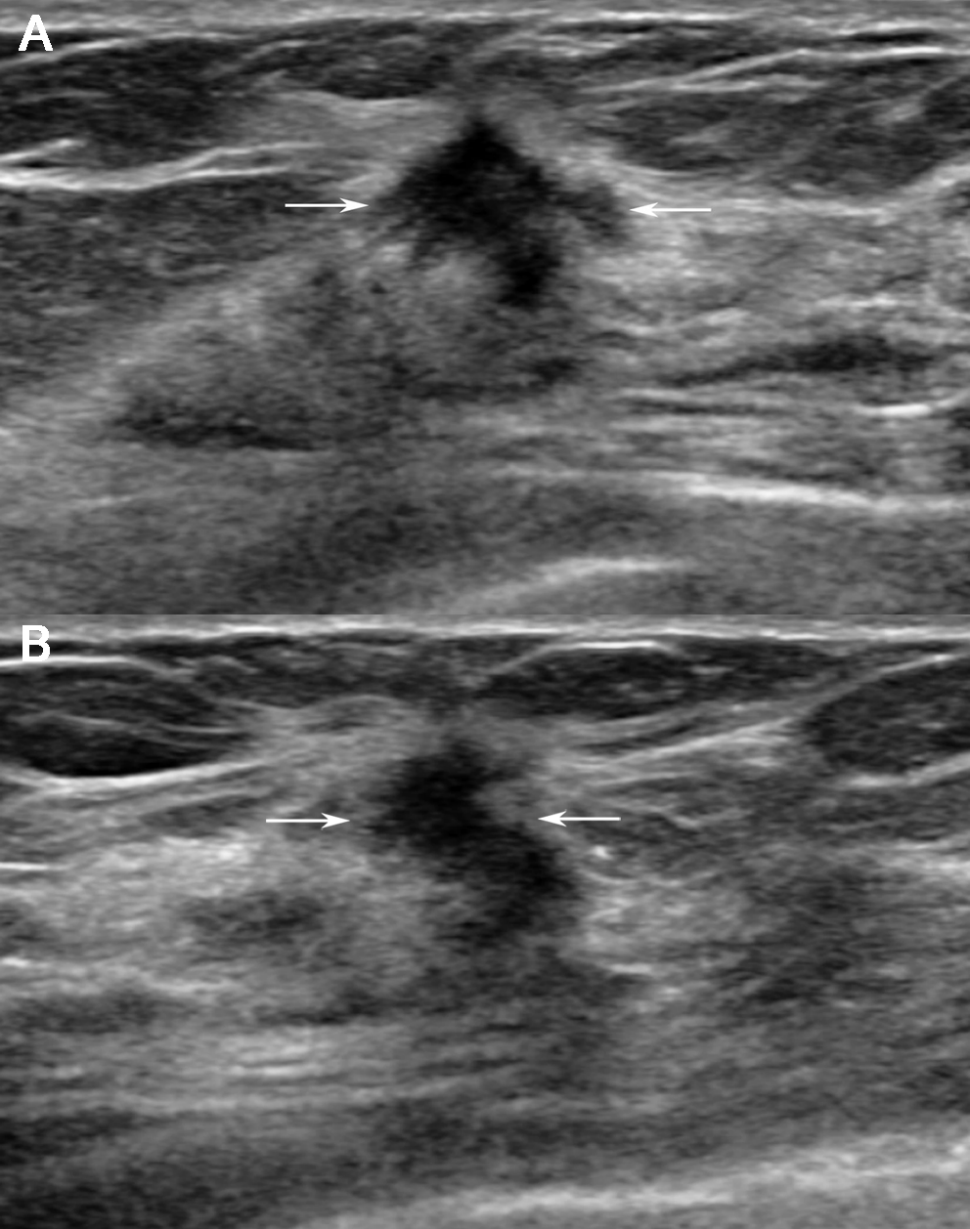
A 63-year-old woman with a recurrence score of 13 (i.e., low risk). US images (A and B) showing an irregularly shaped, hypoechoic mass (arrows) with spiculated margins that is not parallel to the skin (tumour roundness score = 28.45). Surgery confirmed invasive ductal carcinoma with LN metastasis. Immunohistochemistry tests demonstrated PR positivity, HER2 negativity, and negative Ki-67 results.

**Fig 2:**
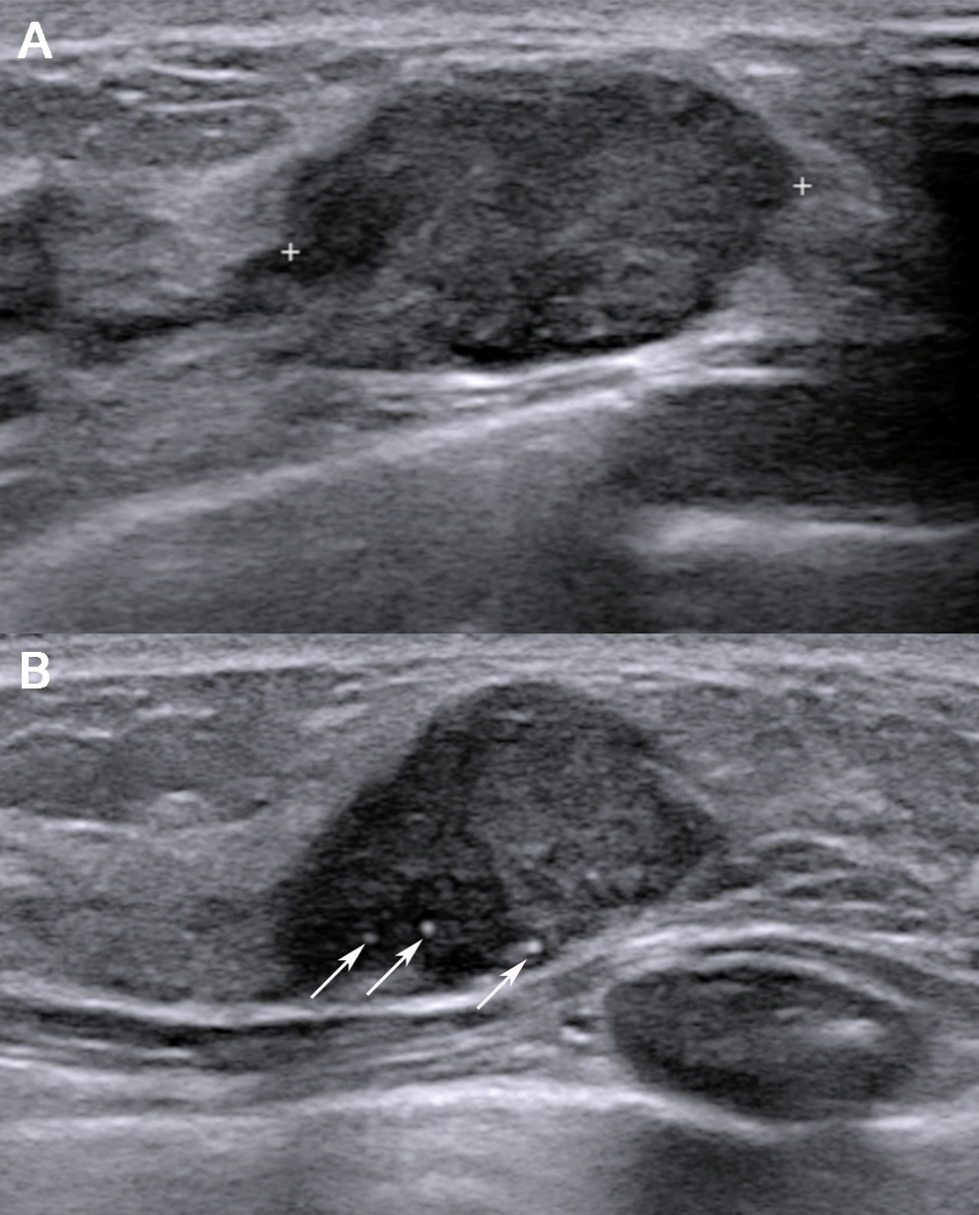
A 38-year-old woman with a recurrence score of 34 (i.e., high risk). US images (A and B) showing an oval-shaped hypoechoic mass with parallel orientation, circumscribed margins, and calcification in the mass (tumour roundness score = 57.45). Surgery confirmed invasive ductal carcinoma with lymphovascular invasion. The immunohistochemistry tests demonstrated PR negativity, HER2 negativity, and a Ki-67 index of 15%.

Of 267 patients, 147 patients (55%) were at low risk of recurrence (Table 1 and Fig 1), 96 patients (36%) were at intermediate risk, and 24 patients (9%) were at high risk (Fig 2) according to the Oncotype DX assay. The 267 patients in this study ranged from 29–70 years of age (mean = 47 years). Tumour size ranged from 4–51 mm (mean = 18 mm). RS did not significantly differ by age or tumour size.

**Table 1:** Summary of the patient characteristics.

Variable		Low risk (RS < 18)	Intermediate risk (RS 18–30)	High risk (RS ≥ 31)	*p* (High vs Non-high)	*p* (Low vs Non-low)
**No. patients**		147 (55)	96 (36)	24 (9)		
**Patient age, y (mean ± SD)**		47.6 ± 8.12	47.4 ± 8.18	46.5 ± 9.45	0.55	0.71
**Tumour size, cm (mean ± SD)**		1.9 ± 0.79	1.9 ± 0.80	2 ± 0.64	0.11	0.85
**Tumour type**	Ductal	129 (88)	87 (91)	22 (92)	0.30	0.71
	Lobular	10 (7)	6 (6)	0 (0)		
	Other	8 (5)	3 (3)	2 (8)		
**LN metastasis**	Negative	102 (69)	70 (73)	17 (71)	0.99	0.55
	Positive	45 (31)	26 (27)	7 (29)		
**Lymphovascular invasion**	Negative	117 (80)	67 (70)	10 (42)	< 0.001	0.004
	Positive	30 (20)	29 (30)	14 (58)		
**Nuclear grade**	Low to intermediate	134 (91)	81 (84)	8 (33)	< 0.001	< 0.001
	High	13 (9)	15 (16)	16 (67)		
**Histologic grade**	Low to intermediate	133 (90)	83 (86)	8 (33)	< 0.001	0.001
	High	14 (10)	13 (14)	16 (67)		
**PR**	Negative	4 (3)	16 (17)	9 (37.5)	< 0.001	< 0.001
	Positive	143 (97)	80 (83)	15 (62.5)		
**P53**	Negative	65 (44)	28 (29)	8 (33)	0.62	0.02
	Positive	82 (56)	68 (71)	16 (67)		
**EGFR**	Negative	147 (100)	95 (99)	21 (87.5)	0.003	0.03
	Positive	0 (0)	1 (1)	3 (12.5)		
**CK56**	Negative	145 (99)	95 (99)	24 (100)	1	0.24
	Positive	0 (0)	1 (1)	0 (0)		
	Unknown	2 (1)	0 (0)	0 (0)		
**Ki-67**	Low	133 (90)	83 (86)	13 (54)	< 0.001	0.02
	High	14 (10)	13 (14)	11 (46)		

RS, recurrence score; SD, standard deviation; PR, progesterone receptor; EGFR, epidermal growth factor receptor; CK, cytokeratin; LN, lymph node. Note—The numbers in parentheses are percentages.

Table 1 summarizes the pathological characteristics. The majority of the tumours were ductal, and lymph node metastases were detected in 29% of patients. RS did not differ significantly in terms of histology or nodal status. Of the pathological variables, lymphovascular invasion, nuclear/histologic grade, PR/EGFR status, and Ki-67 were correlated with RS (*p* < 0.05).

### Association between US features and RS according to the univariate analysis

The US findings according to the BI-RADS lexicon are listed in Table 2. The high-risk group was significantly associated with following features: parallel orientation (*p* = 0.004) and calcification in the mass (*p* = 0.002). Spiculated margins were correlated with low or intermediate RS (*p* = 0.005, Bonferroni-corrected *p* = 0.03). Circumscribed margins and posterior acoustic enhancement were more frequently seen in the high-risk group, however, the difference did not reach statistically significance after Bonferroni correction. RS did not significantly differ according to shape, and irregular shape and hypoechogenicity were the most common findings.

**Table 2:** Association between US features and recurrence scores by univariate analysis.

Variable		Low risk (RS < 18)	Intermediate risk (RS 18–30)	High risk (RS ≥ 31)	*p* (High vs Non-high)	*p*(Low vs Non-low)
**No. patients**		147 (55)	96 (36)	24 (9)		
**Shape**	Oval	28 (19)	21 (22)	7 (29)	0.40	0.69
	Round	13 (9)	7 (7)	3 (13)		
	Irregular	106 (72)	68 (71)	14 (58)		
**Orientation**	Parallel	79 (54)	59 (61)	21 (87.5)	0.004	0.03
	Not parallel	68 (46)	37 (39)	3 (12.5)		
**Margins**					0.01	0.05
	Circumscribed	12 (8)	10 (10)	6 (25)	0.03 [0.15]^a^	0.17 [0.85]
	Indistinct	48 (33)	26 (27)	6 (25)	0.58 [NA]	0.29 [NA]
	Angular	3 (2)	3 (3)	1 (4)	0.49 [NA]	0.70 [NA]
	Microlobulated	26 (18)	26 (27)	9 (38)	0.07 [0.35]	0.03 [0.15]
	Spiculated	58 (39)	31 (32)	2 (8)	0.005 [0.03]	0.04 [0.20]
**Echo pattern**					0.70	0.36
	Complex	3 (2)	0 (0)	0 (0)	1 [NA]	0.26 [NA]
	Hypoechoic	86 (58)	54 (56)	16 (67)	0.39 [NA]	0.98 [NA]
	Isoechoic	10 (7)	5 (5)	0 (0)	0.38 [NA]	0.35 [NA]
	Heterogeneous	48 (33)	37 (39)	8 (33)	0.87 [NA]	0.41 [NA]
**Posterior feature**					0.002	0.01
	No	97 (66)	68 (71)	14 (58)	0.34 [NA]	0.69 [NA]
	Enhancement	9 (6)	10 (10)	6 (25)	0.02 [0.08]	0.04 [0.16]
	Shadowing	37 (25)	14 (15)	1 (4)	0.06 [0.24]	0.01 [0.04]
	Combined	4 (3)	4 (4)	3 (13)	0.07 [0.28]	0.23 [0.92]
**Calcification**	Present	26 (18)	27 (28)	12 (50)	0.002	0.005
	Absent	121 (82)	69 (72)	12 (50)		
**Tumour roundness**	Mean ± SD	35 ± 14.1	45 ± 14.1	50 ± 11.8	0.001	< 0.001
	Range	7.12–76.83	25.80–76.62	31.86–72.39		

Note—The numbers in parentheses are percentages.

^a^ For factors of more than 2 levels (for example, margins), Bonferroni-corrected *p* value was calculated and presented using square brackets.

**Fig 3:**
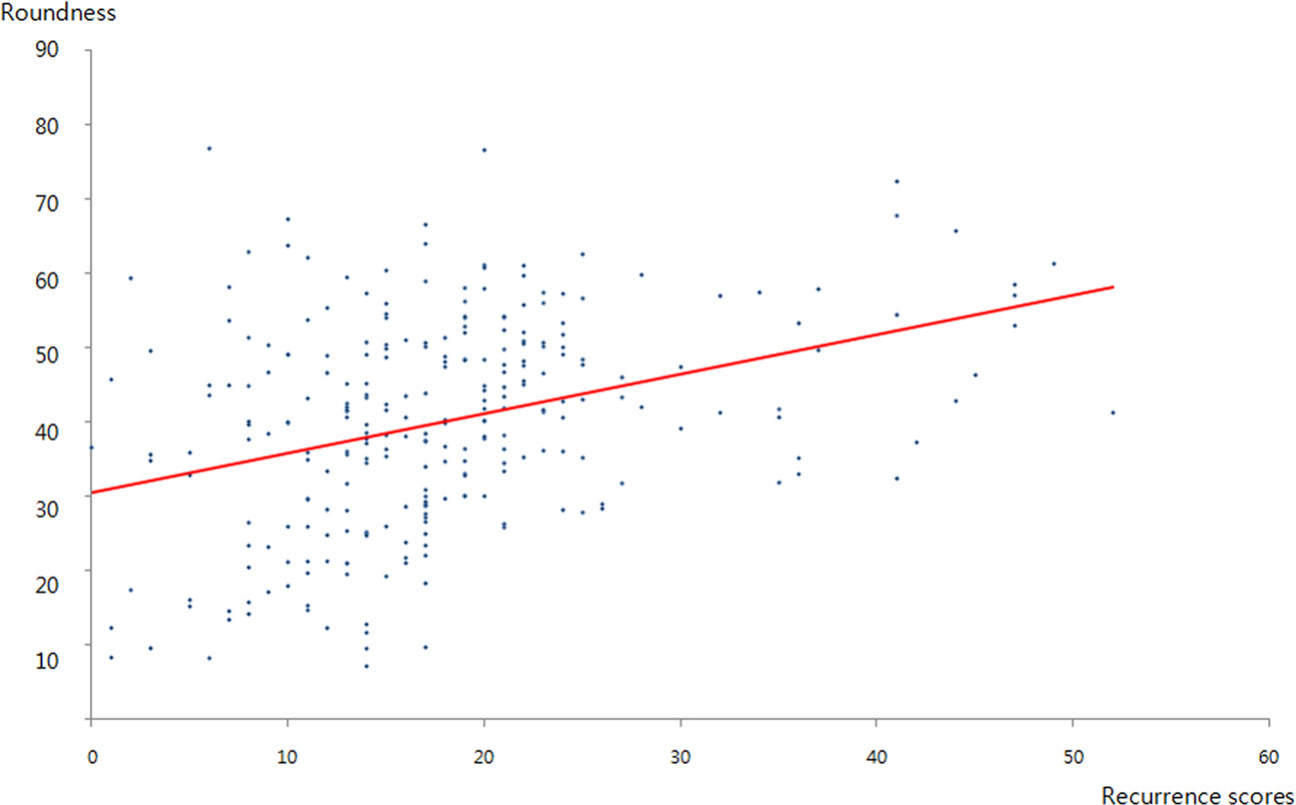
Correlation between the tumour roundness and recurrence score. The correlation plot shows a positive relationship between the tumour roundness and recurrence score (r = 0.349).

When the RS was categorized as low or non-low risk (i.e., intermediate or high), lesion orientation (*p* = 0.03), posterior features (*p* = 0.01), and the absence of calcification in the mass (*p* = 0.005) were associated with the low-risk group. Spiculated margins were associated with low RS, however, the difference did not reach statistically significance after Bonferroni correction (*p* = 0.04, Bonferroni-corrected *p* = 0.20). RS did not significantly differ by shape or echo pattern. Table 2 and Fig 3 show the association between tumour roundness and RS. The mean values for tumour roundness were 35 ± 14.1 (range = 7.12–76.83) in the low-risk group, 45 ± 14.1 (range = 25.80–76.62) in intermediate-risk group, and 50 ± 11.8 (range = 31.86–72.39) in the high-risk group. Tumour roundness and RS were positively correlated (r = 0.349; *p <* 0.05). The interobserver agreement for tumour roundness was substantial, with an intraclass correlation coefficient of 0.65 (95% CI = 0.55–0.72). The intraobserver agreement values for tumour roundness were 0.84 (95% CI = 0.77–0.89) and 0.77 (95% CI = 0.68–0.84) for readers 1 and 2, respectively.

### Logistic regression analysis

**Fig 4:**
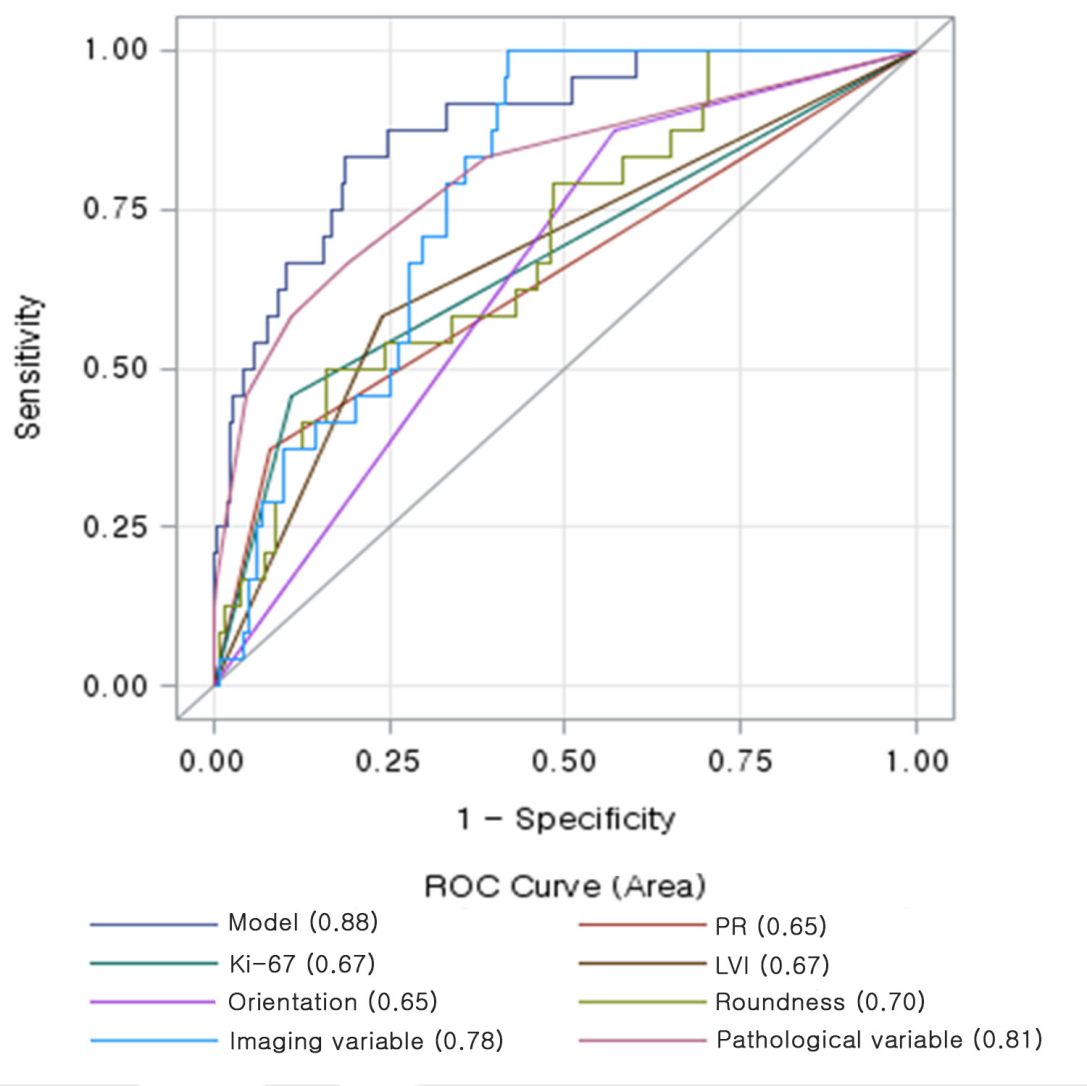
Receiver-operating characteristic curve determined by multivariate logistic regression analysis for distinguishing high recurrence scores from low or intermediate recurrence scores: parallel orientation, tumour roundness, lymphovascular invasion, PR negativity, and high Ki-67 are predictors of recurrence. Data in parentheses indicate the A_z_ values for the each variables.

Variables with *p* < 0.05 on univariate analysis—including lesion orientation, tumour roundness, margins, posterior features, presence of calcification in the mass, lymphovascular invasion, nuclear/histologic grade, PR, EGFR, and Ki-67—were entered as input variables in multivariate analysis in order to distinguish high RS. Multiple logistic regression analysis revealed that parallel orientation, tumour roundness, lymphovascular invasion, PR negativity, and high Ki-67 remained independent variables associated with high RS (Table 3). Among these, PR negativity demonstrated the highest odds ratio (OR = 7.14; 95% CI = 2.22–23.81) for predicting high RS on the multivariate analysis. The area under the ROC curve (A_z_) for the pathological variables—including lymphovascular invasion, PR negativity, and high Ki-67—was 0.81 for distinguishing high RS from low or intermediate RS. The Az for the imaging variables was 0.78 and increased to 0.88 in the resulting model that included parallel orientation, tumour roundness, lymphovascular invasion, PR negativity, and high Ki-67 as predictors (Fig 4).

**Table 3:** Multivariate analysis of factors associated with high recurrence scores.

Variable	Odds ratio	95% CI	*p*
Parallel orientation	5.53	1.46–30.30	0.02
Tumour roundness (per 10 increase)	1.70	1.12–2.58	0.01
Lymphovascular invasion	4.98	1.80–14.68	0.002
PR negativity	7.14	2.22–23.81	0.001
High Ki-67	4.56	1.55–13.50	0.005

**Fig 5:**
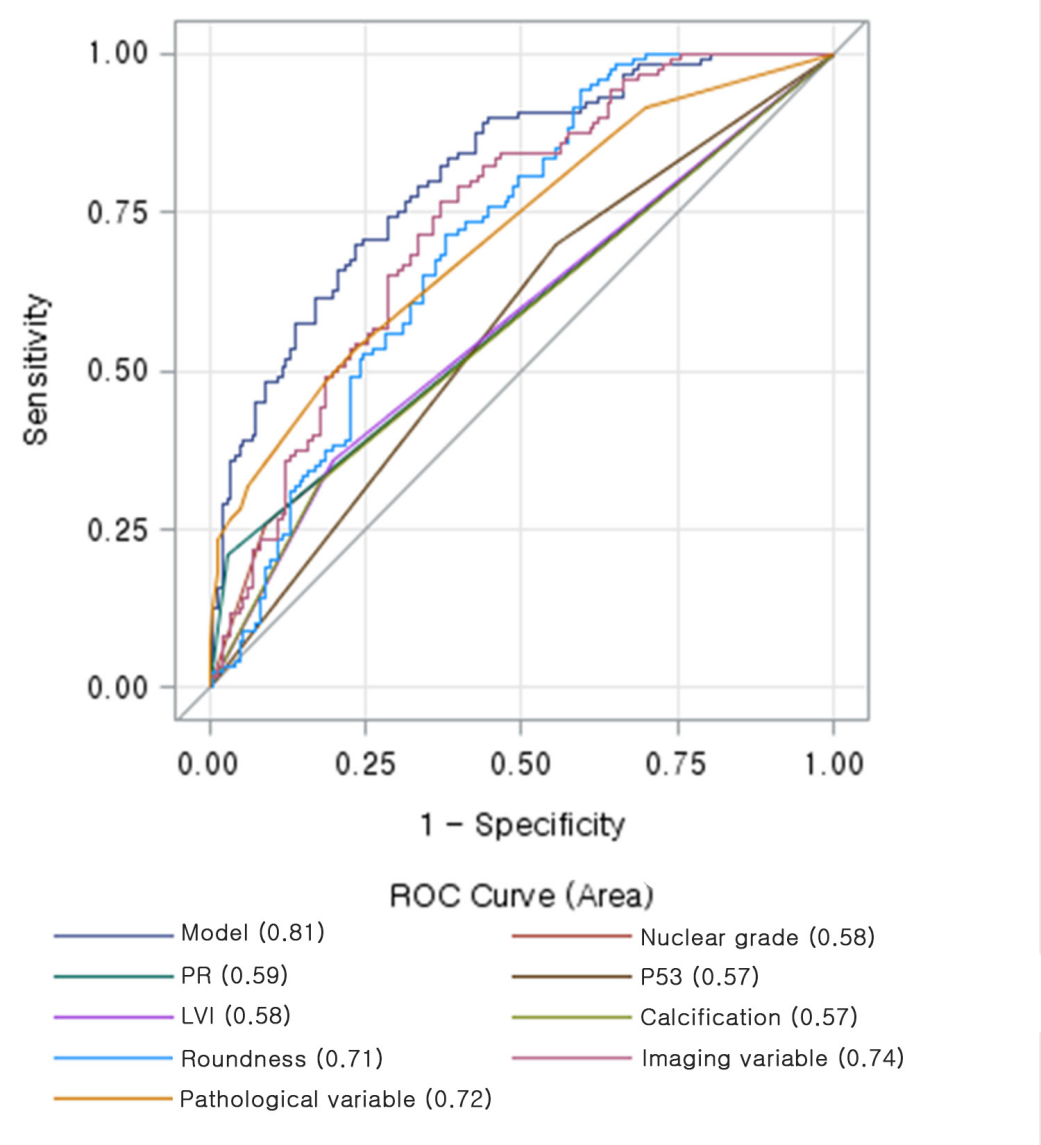
Receiver-operating characteristic curve determined by multivariate logistic regression analysis for distinguishing low recurrence scores from high or intermediate recurrence scores: tumour roundness, absence of calcification in the mass, lymphovascular invasion, PR positivity, nuclear grade, and p53 are predictors of recurrence. Data in parentheses indicate the A_z_ values for the each variables.

We also performed multiple logistic regression analysis to identify independent predictors of low RS. Of all variables, the absence of calcification in the mass, tumour roundness, no lymphovascular invasion, PR positivity, low nuclear grade, and p53 positivity remained independent variables associated with low RS (Table 4). The A_z_ values for the imaging and pathological variables were 0.74 and 0.72, respectively. A_z_ increased to 0.81 in the resulting model that used all independent variables (Fig 5).

**Table 4:** Multivariate analysis of factors associated with low recurrence scores.

Variable	Odds ratio	95% CI	*p*
Absence of calcification in the mass	2.18	1.10–4.33	0.03
Tumour roundness (per 10 decrease)	1.90	1.50–2.41	< 0.001
No lymphovascular invasion	2.35	1.22–4.52	0.01
PR positivity	9.35	2.75–32.26	< 0.001
Low nuclear grade	2.74	1.16–6.47	0.02
p53 positivity	2.66	1.39–5.08	0.003

## Discussion

Several commercialized gene expression assays are currently available, and there is an increasing number of efforts to incorporate these results for predicting a patient’s outcome and treatment response [17–19]. Oncotype DX is the prognostic profiling multigene expression assay that is included in the National Comprehensive Cancer Network (NCCN), American Society of Clinical Oncology (ASCO), and St. Gallen’s guidelines for breast cancer [20–22].

The main strength of our current study is that it indicates the US features that could distinguish high RS from low or intermediate RS, as well as low RS from high or intermediate RS, in patients with ER-positive breast cancer. Although several previous studies have assessed the associations between imaging features and the results of multigene expression assays, these reports only analyzed the imaging features that predict high RS [13] or low RS [14]. Ashraf et al. [13], in their preliminary study, showed that intrinsic imaging phenotypes exist for breast cancer using a multiparametric quantitative imaging vectors and correlate with recurrence likelihood.

Our multivariate analysis found that parallel orientation (OR = 5.53; *p* = 0.02) and tumour roundness (OR = 1.70 per 10 increase in the roundness value; *p* = 0.01) are independent variables associated with high RS on Oncotype DX. We also found that the tumour roundness (OR = 1.90 per 10 decrease in the roundness value; *p* < 0.001) and the absence of calcification in the mass (OR = 2.18; *p* = 2.18) are independent predictors associated with low RS. Tumour roundness is a quantitative measure that provides information about the morphological features of a tumour. As the tumour becomes closer to being a perfect circle, the roundness score increases up to a score of 100. Bae et al. [12] reported the correlation between tumour roundness (as measured using magnetic resonance imaging) and immunohistochemical biomarkers of breast cancers. In that study, tumour roundness demonstrated an inverse correlation with the ER score and a positive correlation with the Ki-67 index, and triple-negative tumours demonstrated a higher tumour roundness score in comparison with the other subtypes. In our current study, the mean tumour roundness score was significantly higher in high-RS group and was an independent variable associated with high-RS group. Our current results are similar to those of that previous study, in that biologically aggressive tumours demonstrated high tumour roundness and all patients with high RS demonstrated tumour roundness scores > 31 (Table 2). In other words, 26% of patients (71 of 267 patients) that had a tumour roundness score < 31 in our present study could skip chemotherapy.

In our current analyses, posterior acoustic enhancement and circumscribed margins were more frequently seen in the high-risk group, although the difference did not reach statistically significance after Bonferroni correction. High-grade carcinomas are more likely to demonstrate posterior acoustic enhancement [10,23,24]. Because high-grade tumours—which have higher mitotic rates and higher cellularity—may have more uniform internal interfaces and/or go through internal necrosis [10], they demonstrate less attenuation to US waves in comparison with the surrounding tissue, thereby leading to brighter signals posterior to the tumours (i.e., posterior enhancement). Circumscribed margins are also more common in high-grade tumours, and spiculation is more frequently noted in low-grade tumours [24]. High-grade carcinoma, therefore, may paradoxically share the imaging features of a benign breast mass. This could also apply to ER-positive breast cancer. Similar results were found regarding lesion orientation. Conventionally, parallel orientation suggestive of containment in 1 tissue plane is a known indicator of a benign process [25]. However, in our present analyses, parallel orientation was found to be an independent predictor associated with high RS.

And in this study, the high-risk group was associated with the presence of calcification in the mass. This finding agrees with a previous study by Yepes et al [14]. They reported that a mass with pleomorphic microcalcifications on mammography may predict an intermediate to high RS in patients with stage I-II ER positive, HER2 negative, and lymph node negative invasive breast cancer. Calcifications are the result of necrotic cellular debris from the duct lumen of cancer and have been associated to a poor prognosis.

Clinical variables such as tumour grade or Ki-67 could reflect tumour biology and have influence on outcome of breast cancer including prognosis and tumour recurrence. Therefore, pathologic data, including immunohistochemistry results, were also evaluated to determine the relationship with RS on Oncotype DX. In our present study, PR negativity, lymphovascular invasion, high nuclear/histologic grade, and high Ki-67 were found to be correlated with high RS. Among these, PR negativity demonstrated the highest odds ratio for predicting high RS by multivariate analysis (OR = 7.14). Previous studies have shown that the loss of PR expression is associated with a more aggressive subset of ER-positive breast cancer, higher risk of relapse, and poorer overall prognosis [26–28]. The absence of PR expression is believed to reflect nonfunctional ER and resistance to hormonal therapy [28].

Because the tissue samples used in gene expression assay were obtained from a very small portion of the breast tumour, the data may not reflect the characteristics of the whole breast tumours because of their intrinsic heterogeneous nature. In this context, the radiogenomic analysis has strength because it can provide comprehensive *in vivo* information about the entire lesion. The current cost of the Oncotype DX test is about $4,000. Although several studies indicate that Oncotype DX is cost-effective for assessing lymph node-negative, ER-positive breast cancer patients [29–31], its high cost may be a barrier for widespread use. In our present study, the area under the ROC curve increased from 0.81 to 0.88 when the imaging variables were added to distinguish high RS from low or intermediate RS. Therefore, if we could predict the risk of recurrence using imaging features in combination with pathological data obtained during routine clinical practice, we could identify ER-positive breast cancer patients who would benefit from adjuvant chemotherapy or endocrine treatment.

This study had several limitations. First, this was a retrospective study, although we did enroll a quite large number of consecutive patients from 2 institutions. Second, only a small portion of our study patients (9%) demonstrated high RS. However, several previous studies also report a relatively low proportion of high RS patients in comparison with low or intermediate RS patients [13,14,32]. Third, we only evaluated a limited number of static US images and real-time evaluation was not possible. Next, observer variability may be an issue because we evaluated tumour roundness using representative images. However, each radiologist independently measured tumour roundness, and the interobserver agreement was substantial. The intraobserver agreement values for the measurement of tumour roundness exceeded 0.75, which indicates high reliability. Automatic lesion segmentation could also be applied to our software program. And, as we did not investigate whether patients had a history of prior biopsy or surgery to the same breast or prior history of breast cancer with treatment, women with an increased risk might have been included. Lastly, one of the major limitations was that we evaluated the relationship between imaging features and the recurrence score, not the actual recurrence in the patient. Because the patients in this study have been recruited since 2010 and ER-positive breast cancers tend to recur slowly over a period of several years, the follow-up period was not sufficient to assess the actual recurrence. It is expected to be able to evaluate the correlation between the imaging features and the actual recurrence after a lapse of time. To confirm our results, further prospective studies with a larger number of patients are warranted.

In conclusion, tumour roundness and parallel orientation are independent variables that may predict a high RS in patients with ER-positive, HER2-negative breast cancer. Moreover, if these results were integrated with pathological data—such as PR negativity, lymphovascular invasion, and high Ki-67—valuable information could be obtained for differentiating patients with ER-positive, HER2-negative breast cancer who would benefit from adjuvant chemotherapy and endocrine treatment, especially if Oncotype DX testing is unavailable.

## Supporting Information

S1 File Sample details and analysis results. (XLSX)
